# The Accuracy of Bone Assessment Distal to Lower Second Molars Using Panoramic Radiography: A Systematic Review and Meta-Analysis

**DOI:** 10.3390/dj12030073

**Published:** 2024-03-11

**Authors:** Hassan Assiri, Albert Estrugo-Devesa, Xavier Roselló-Llabrés, Sonia Egido-Moreno, José López-López

**Affiliations:** 1 Department of Odontostomatology, Faculty of Medicine and Health Sciences (Dentistry), University of Barcelona, 08970 Barcelona, Spain; halmuawad@kku.edu.sa (H.A.); albertestrugo@ub.edu (A.E.-D.); xavierroselloll@ub.edu (X.R.-L.); soniaegido@ub.edu (S.E.-M.); 2 Department of Diagnostic Science and Oral Biology, Faculty of Dentistry, King Khalid University, Abha 61421, Saudi Arabia

**Keywords:** tooth impaction, orthopantomography, third molar, marginal bone loss

## Abstract

Panoramic radiography (OPG) evaluates mandibular third molar impaction (MTMI). This systematic review aimed to investigate the diagnostic accuracy of OPG in detecting bone loss distal to the lower second molars. The associated bone loss with different impaction positions and the most prevalent positions of MTMI were investigated as secondary outcomes. In January 2023, PubMed, Scopus, and Cochrane were searched to identify studies published between January 2012 and January 2023. Two examiners blindly selected the eligible studies for data extraction and quality assessment. Of 427 studies, 8 were suitable for data extraction. All studies reported bone loss distal to the second molar using OPG, ranging from 4.9 to 62.9%. The most frequent position of MTMI is mesioangular. The distal bone loss in the vertical and horizontal positions is statistically significant compared to typically positioned third molars and those that are fully erupted or impacted, but in a normal orientation (*p*-value 0.005 and 0.02, respectively). Bone loss was not statistically significant in the mesioangular position compared to other impacted positions (*p*-value 0.14). The risk of bias ranges between 66 and 88%. Despite its limitations, OPG is still considered a valuable tool to assess bone loss distal to the lower second molar in cases of an impacted mandibular third molar.

## 1. Introduction

The term impaction is derived from the Latin term impactus, which generally describes the status in which a wholly developed tooth fails to achieve its normal physiologic position in the corresponding eruption time [1]. Therefore, the impacted tooth is seen to be completely or partially unerupted and situated against another tooth, bone, or tissue, so that eruption is unlikely [2]. Accordingly, Andreasen et al. [3] described impaction as a cessation of the eruption of a tooth caused by a clinically or radiographically recognizable physical hindrance within the eruption pathway or by the ectopic position of the tooth. The mandibular third molar (MTM) is considered the most impacted tooth in the oral cavity. It is expected to adopt its physiological and anatomical position as early as 16 or as late as 18 to 20 years [4].

The maxillary canine is the second most frequently impacted tooth, after the third molar [5]. Regarding the causes of mandibular third molar impaction, several factors lead to its occurrence, categorized as systemic or local factors. Systemic factors include genetics and endocrinal insufficiency. Local factors contribute more commonly to tooth impaction, and can include the ectopic position of the tooth germ, insufficient tooth space, the presence of a supernumerary tooth, and a disrupted eruption pathway [6]. The occurrence of mandibular third molar impaction (MTMI) ranges from 9.5% to 68.6% [7]. Among all impacted teeth, it is found to occur in about 24.4% of the population [8]. Most studies have reported no sexual predilection [9,10,11,12]. However, some studies have postulated higher frequency in females [13,14,15].

Regarding radiographic diagnosis, orthopantomography (OPG) is a diagnostic tool frequently used in dental practice. It provides a broad view of the maxillofacial structures, including dental arches, alveolar bones, temporomandibular joints, and facial bones, and depicts them in one image [16]. Managing MTMI poses complications such as postoperative bleeding, alveolitis, mandibular angle fracture, transient inferior alveolar nerve damage, and a permanent loss of nerve sensation. Although MTMI causes several complications, OPG can assist in avoiding them [17,18]. OPG can initially indicate the overall status and any possible occurrence of complications. MTMI can exert effects on the surrounding tooth and bone structures. The abnormal anatomical relationship between the MTMI and the adjacent tooth can lead to food accumulation, plaque formation, and microbial proliferation, increasing the risk of periodontal degeneration [19,20]. In this regard, MTMI is found to be associated with a higher prevalence of periodontal disease on the distal aspect of the second molar [21].

Distal caries on the second molar occurs mainly on the distocervical area in mesioangular impaction [22,23]. For diagnosis, two-dimensional radiography, including OPG, has been considered the standard imaging tool for different aspects of the oral cavity. It provides supportive information to facilitate proper decision-making [24]. However, cone beam computed tomography (CBCT) is also available, having been introduced into dental practice over two decades ago. It is a three-dimensional imaging tool considered more accurate in revealing information than conventional radiography. In periodontology, it plays a significant role in determining furcation involvement and guides apical surgical intervention [25]. Although CBCT is considered superior to two-dimensional techniques in terms of the information provided and its accuracy, its use depends on weighing these benefits against the risks. Therefore, CBCT use must be justified to avoid unnecessary radiographic exposure [26]. Regarding the management of MTMI, several studies have investigated prophylactic removal to prevent the effects on neighboring teeth and periodontal tissues [27,28,29]. Vrancx et al. concluded, in their systematic review, that the study findings indicate that compelling variables relating to patients and surgery support the prompt extraction of the third molar, ideally before the age of 25, mainly to prevent long-term morbidity and nerve problems [30].

As a routine diagnostic tool, OPG is used to visualize MTMI [16]. Although it has limitations in magnification, superimposition, and the inadequate sharpness of delicate structures, it assists in mapping out the position of MTMI and providing insight into its associated pathologies and the effect on nearby structures [31]. Several studies have reported the affordability of two-dimensional radiography, including periapical and OPG, in periapical lesion detection and dental implant follow-up [32,33]. Regarding affordability, we aimed to perform this systematic review to investigate the diagnostic accuracy of OPG for the detection of bone loss distal to the lower second molars. The bone loss associated with different impaction positions and the most prevalent positions of MTMI were investigated as secondary outcomes.

## 2. Materials and Methods

This review was conducted according to the guidelines of Preferred Reporting Items for Systematic Review and Meta-Analysis (PRISMA) and guidance from the Center for Reviews and Dissemination (CRD) for undertaking a systematic review in healthcare [34,35]. The protocol is registered with PROSPERO (International Prospective Register of Systematic Reviews) with the ID number CRD42023476404 [36]. The review question was designed according to the PICO (Population or Problem, Intervention or Exposure, Comparison, Outcome) element [37]. Regarding the problem specification, the research question was, “What is the diagnostic accuracy of panoramic radiography in detecting bone loss distal to the lower second molar in patients having third molar impaction, and what is the status of bone loss with regard to different impaction positions, and what is the most prevalent position reported using OPG”?

P: Patients with impacted mandibular third molar.
I: OPG.
C: cone beam computed tomography (CBCT) or clinical measurements, including intrasurgical measurements if they are used in the included studies.
O: Status of bone loss distal to lower second molar in cases of third molar impaction.

The inclusion and exclusion criteria were preidentified. Then, the included studies were analyzed to answer the research question.

### 2.1. Inclusion Criteria

- Original studies;
- Studies reporting the effects of impacted mandibular molar on the bone distal to the lower second molar using panoramic radiography;
- Studies comparing OPG to other imaging tools for assessing bone loss distal to the lower second molar;
- Studies reporting types of impaction associated with bone loss on the distal aspect of the lower second molar;
- Studies published from January 2012 to January 2024.

### 2.2. Exclusion Criteria

- Case reports;
- Narrative reviews;
- Book chapters;
- Languages other than English and Spanish.

### 2.3. Search Strategy

The search strategy was designed for each chosen database using keywords. In January 2023, PubMed, Scopus, and Cochrane databases were searched. In PubMed, we explored using the following terms: (tooth impaction AND third molar) AND panoramic radiograph. In Scopus, we searched according to TITLE-ABS-KEY (tooth impaction), AND TITLE-ABS-KEY (third molar) AND TITLE-ABS-KEY (panoramic radiography). In Cochrane Library, we searched according to the following search term combination: tooth impaction (word variations: ti, ab, kw) AND third molar (word variations: ti, ab, kw) AND panoramic radiography (word variations: ti, ab, kw). PubMed, Scopus and Cochrane databases were searched to identify studies that used panoramic radiography to assess the bone loss distal to the lower second molar associated with lower third molar impaction. The search was conducted to retrieve the relevant studies based on the PRISMA flow chart for reporting a systematic review.

### 2.4. Study Retrieval

The studies retrieved from the databases were subjected to a duplicate check via RefWorks^®^. Then, two authors (SEM and AED) blindly screened the results for relevance based on the inclusion and exclusion criteria. After that, the studies that met the eligibility criteria were assigned for full-text reading. Where there was uncertainty, discussions were conducted between the authors (H.A., AED, SEM, XRLL, and JLL) to agree on whether to include or exclude a study according to the predefined inclusion and exclusion criteria. Finally, the selected studies were assigned for data extraction and analysis.

### 2.5. Data Collection

One author (HA) collected and extracted data from the selected articles. Then, two authors (S.E.-M. and A.E.-D.) discussed the checking of the data needed for further analysis. The data were extracted according to the following criteria: author, year of publication, diagnostic imaging tool, study design, study type, sample size, quality assessment tool, and risk of biased assessment.

### 2.6. Risk of Biased Assessment

Each study was evaluated using the recommended checklist based on the study type. Prevalence studies were evaluated according to the Joanna Briggs Institute (JBI) Critical Appraisal Checklist [38]. Another measure adopted to avoid biased assessment was having two authors (SEM and AED) who did not know each other blindly review all the studies. All authors (HA, AED, SEM, XRLL, and JLL) approved the grading system before commencing the critical appraisal to standardize the criteria of biased assessment. The risk of bias is considered high if less than 40% of the checklist is fulfilled, moderate if 40% to 80% is fulfilled, or low if more than 80% is fulfilled.

### 2.7. Statistical Analysis

The Review Manager 5.4 program was used to analyze the data previously recorded in an Excel table by the author (SEM). The summary measures were quantified as percentages and numbers to compare the corresponding mandibular third molar position associated with bone loss. Forest plots were performed to graphically represent the vertical, mesioangular, and horizontal positions of the mandibular third molar on OPG, with a 95% confidence interval (CI). The *p*-value (*p*) = 0.05 was used for the significance level. Heterogeneity was assessed using the I2 test. The kappa test was used to determine the correlation of bone loss between CBCT and OPG [39]. Kappa values were defined as follows: no agreement if < 0; slight agreement if between 0.00 and 0.20; fair agreement if between 0.21 and 0.40; moderate agreement if between 0.41 and 0.60; substantial agreement if between 0.61 and 0.80; and almost perfect agreement if between 0.81 and 1.00. 

## 3. Results

### 3.1. Study Selection

**Figure 1:**
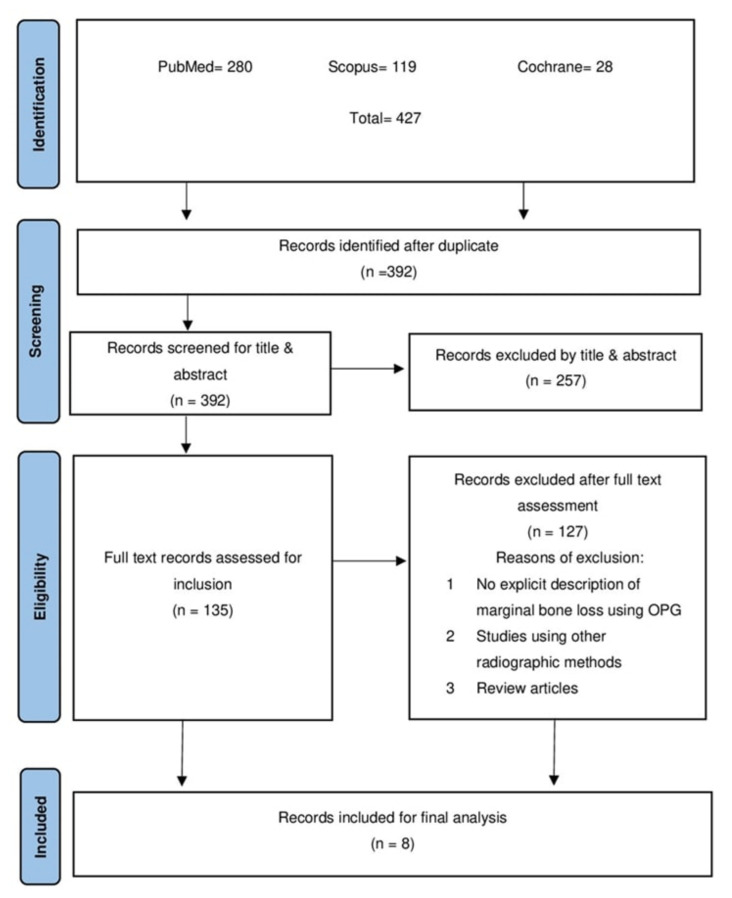
The PRISMA flow chart for reporting systematic reviews.

Searching the three databases yielded 427 articles: 280 from PubMed, 119 from Scopus, and 28 from Cochrane. All subsidies were imported into the RefWorks ^®^ database for managing references to check for duplicates, and 392 were subjected to blind title and abstract screening by two authors after the duplicate checks were performed. Then, a set of 135 studies was considered for full-text screening. Finally, eight studies were suitable for data extraction, analysis, and a risk of bias assessment. The PRISMA flow chart indicates the process of study retrieval (Figure 1).

### 3.2. Study Characteristics

Observational analytical studies that investigated the marginal bone loss using OPG versus CBCT conducted by Días et al. [40] and Matzen et al. [41] were evaluated using the Joanna Briggs Institute (JBI Critical Appraisal Checklist, a checklist used for prevalence studies [38]. Similarly, the prevalence cross-sectional studies by Gupta et al. [42], Tai et al. [43], Savitri et al. [44], Ye et al. [45], Altan et al. [46], and Shumar et al. [47] were assessed using the same tool (JBI) (Table 1).

Table 2 details the extracted data analysis and the quality assessment for each study. According to our evaluation, Dias et al. [40] fulfilled 100% of the checklist criteria, indicating a low risk of bias. Matzen et al. [41] fulfilled approximately 88% of the checklist criteria, indicating a low risk of bias. Furthermore, Tai et al.’s study [43] was regarded as having a low risk of bias since the study met around 88% of the checklist criteria. Gupta et al. [42], Savitri et al. [44], Ye et al. [45], Altan et al. [46], and Shumar et al. [47] met only 77% of the checklist criteria and hence were regarded as having a moderate risk of bias.

### 3.3. Descriptive Analysis

This systematic review indicated 6631 lower third molars in the selected studies. The meta-analysis assessed the robustness of the collected evidence in analyzing this number of teeth. The frequency of bone loss distal to the lower second molar ranged from 4.9% to 62.9%. Five studies provided information on the number of patients (i.e., Dias et al. [40]; Gupta et al. [42]; Tai et al. [43]; Ye et al. [45]; and Altan et al. [46]), with a total of 3512 patients. On the other hand, Matzen et al. [41] and Savitri et al. [44] did not provide data on the number of patients. Although Shumar et al. [47] included 741 patients, this number also included upper molars, which were not the object of study in this review.

Only Dias et al. [40], Gupta et al. [42], and Altan et al. [46] included information about sex. Out of 1424 participants, 51.1% (*n* = 728) were females and 48.9% (*n* = 696) were males. Matzen et al. [41], Tai et al. [43], Savitri et al. [44], and Ye et al. [45] did not provide data about participants’ sex. Of Shumar et al.’s [47] patients, 63.1% (*n* = 467) were women and 36.9% (*n* = 274) were men, but the study included upper molars.

Five studies reported the participants’ age (Dias et al. [40], Matzen et al. [41], Gupta et al. [42], Ye et al. [45], and Altan et al. [46]), with an average age of 27.08 ± 2.17 years.

As for the position of the lower third molars, six studies—Dias et al. [40], Gupta et al. [42], Tai et al. [43], Savitri et al. [44], Ye et al. [45], and Altan et al. [46]—provided data on 5341 wisdom teeth. The most prevalent position was mesioangulated (49.8%; *n* = 49.8), followed by vertical (24.3%; *n* = 24.3), horizontal (19%; *n* = 1015), inverted (4%; *n* = 212), distoangular (2%; *n* = 109), and transverse (0.02%; *n* = 1). The remainder were unspecified (0.8%; *n* = 43).

Matzen’s study dichotomizes the wisdom teeth into mesioangular/horizontal and others [41]. It finds the mesioangular/horizontal position to be more prevalent. Shumar’s study informs us of the position but includes the upper wisdom teeth in the data and does not differentiate between maxillary and mandibular wisdom teeth. The predominant position is found to be mesioangular, followed by distoangular and vertical [47].

**Figure 2:**
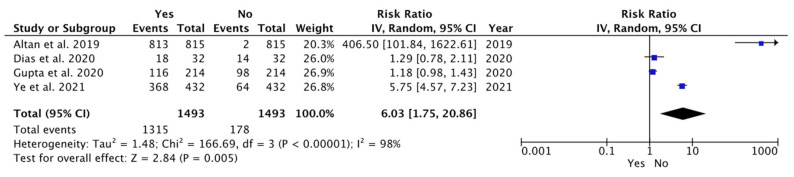
Meta-analysis of the studies according to the vertical positions associated with bone loss [40,42,45,46].

Based on our meta-analysis, the forest plot analyzed the status of vertical impactions associated with bone loss in the different studies. It indicated that bone loss was statistically significant in the vertical position compared to other impaction positions (weighted mean difference (WMD): 6.03; 95% CI: 1.75 to 20.86, *p* = 0.005; and heterogeneity: I^2^: 98%, *p* = 0.005) (Figure 2). In systematically reviewing the literature, one of the aims was to investigate whether the bone loss associated with a particular impaction position was statistically significant among the different studies compared to other impaction positions.

**Figure 3:**
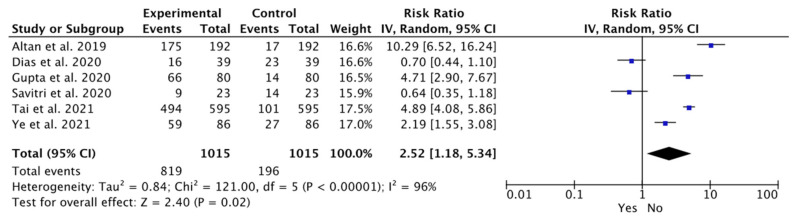
Meta-analysis of the studies according to the horizontal position associated with bone loss [40,42,43,44,45,46].

Similarly, the plot shows the status of horizontal impaction associated with bone loss in the different studies. It indicates that bone loss was statistically significant in the horizontal position among various studies compared to other impaction positions (WMD: 2.52; 95% CI: 1.18 to 5.34, *p* = 0.02; and heterogeneity: I^2^: 96%, *p* = 0.02) (Figure 3).

**Figure 4:**
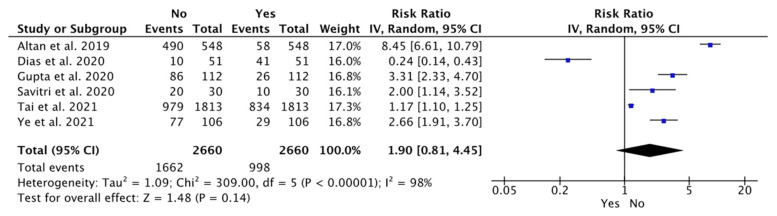
Meta-analysis of the studies according to the mesioangular position associated with bone loss [40,42,43,44,45,46].

However, the bone loss was not statistically significant in the mesioangular position among the different studies compared to other impaction positions (WMD: 1.90; 95% CI: 0.81 to 4.45, *p* = 0.14; and heterogeneity: I^2^: 98%, *p* = 0.14) (Figure 4). The correlation between OPG and CBCT was observed in two studies with six observers [40,41].

Both studies presented the results of using CBCT and OPG in evaluating bone loss. Matzen et al.’s study [41] comprised four individual observations, while in the work by Dias et al. [40], two observers assessed the images. Accordingly, our analysis indicated that kappa values vary between 0.368 and 0.579, interpreted as fair agreement and moderate agreement, respectively.

## 4. Discussion

Bone loss distal to the lower second molar is considered one of the possible complications of an impacted third molar. This systematic review analyzed the bone loss distal to the second molar when associated with an impacted third molar. Dias et al. [40] compared OPG and CBCT images for 70 patients, looking at the bone loss assessment distal to the second molar. Bone loss is manifested in 62.9% of OPG compared to 80% of CBCT. On OPG, the team relied on the integrity of the alveolar bone crest to determine the presence or absence of bone loss. They further classified the bone loss severity into slight, moderate, or severe, based on the loss level from the coronal third of the second molar up to the apical third. Additionally, the intra-examiner agreement for classifying the presence or absence of bone loss reached 82.5% in their study.

Furthermore, the quantitative bone loss was measured only in the CBCT scans because of limitations in panoramic radiography. This reflects the fact that CBCT is a more reliable and accurate means of performing bone measurements compared to OPG. Similarly, Matzen et al. [41] compared the pathological findings seen on orthopantomography OPG and CBCT, including marginal bone loss on the distal surface of the second molar. On OPG, they considered bone loss to be evident when it exceeded 3 mm. All four observers, who were trained oral radiologists, found that mesioangulated and horizontally positioned third molars, as assessed in OPG, were strongly associated with marginal bone loss as observed via CBCT. This finding agrees with those of Dias et al. [40] regarding the accuracy of CBCT compared to OPG when assessing marginal bone loss. Consequently, it is obvious that OPG considerably underestimates the third-molar-related negative impact on the periodontium compared to CBCT.

Gupta et al. [42] investigated the occurrence of periodontitis adjacent to the lower second molar in the presence of an impacted third molar on panoramic radiographs and found that it accounts for 39%. Horizontal bone loss occurs more than vertical bone loss. The vertical bone loss was more evident with the mesioangular impaction. However, they did not specify the measurement method for bone loss on panoramic images, and the sample size included in the study needed to be revised. Tai et al. [43] studied the association of mandibular third molar impaction with dental and periodontal lesions in the adjacent mandibular second molar on panoramic radiographs. Their study concluded that the distal bone loss for the second molar accounts for 35.30% of the total and is seen more frequently with mesioangular impaction. They also classified the bone loss severity concerning the distal aspect of the second molar, for which the most severe form manifests, including the total distal aspect of the distal root and the furcation area. The mild form of bone loss is < 2/3 the distal root length. Their findings agree with those of Gupta et al. [42] regarding increased bone loss with age. Savitri et al. [44] described pathological abnormalities associated with horizontal and mesioangular lower-mandibular third molar impaction using panoramic images. A loss of interalveolar bone between the third and second molar represents the pathology seen most frequently with horizontal impaction, compared to the mesioangular type.

Ye et al. [45] evaluated the pathologies related to the mandibular impacted third and second molar on panoramic radiographs. Marginal bone loss distal to the lower second molar was regarded as a pathology and accounted for 14.81%. It was their subjects’ third most common pathology after pericoronitis and dental caries.

Altan et al. [46] assessed the impact of different angulations of the impacted mandibular third molar on the presence of other pathologies using panoramic radiography. The bone loss from the distal surface of the mandibular second molar accounted for 4.9%.

Shumar et al. [47] investigated the occurrence of different pathologies in impacted third molars in the Yemeni population. They concluded that in the case of lower third molar impaction, the distal bone loss related to the second molar manifested in about 6.8% of the study’s participants. The measurement method for bone loss and inter-observer variability was not stated in this study.

We were able to refer to previously published studies that used OPG to report the prevalence of impaction and the condition of associated pathologies, including marginal bone loss from the distal aspect of the lower second molar. However, the linear measurement needed to be explicitly explained.

The quality assessment for the studies included in this review was based on the extent to which the studies met the checklist criteria. According to our interpretation, studies were considered to have a low risk of bias when they met all or most of the requirements. However, some should have explicitly reported the sampling method and the sample calculation. For example, Gupta et al.’s study [42] needed to clarify whether the sample frame was appropriate for the target population. In addition, it needed to explain whether the participants were sampled appropriately and adequately. Similarly, the studies by Savitri et al. [44], Ye et al. [45], Altan et al. [46], and Shumar et al. [47] were regarded as unclear in terms of the sampling method and whether the sampled frame addressed the target population.

Although all the studies reported marginal bone loss distal to the lower second molar using panoramic radiography, it was not explicitly explained how the linear measurements were conducted to determine the degree of bone loss.

In terms of managing mandibular third molar impaction, authors conducted follow-up assessments after a mean period of five years. They found that in cases where the impacted third molar is located close to the alveolar nerve, and there is no peri-apical infection of the inferior third molars, leaving the crown and the bone distal to the second molar completely healed is preferred. In a few cases, however, a second surgery may be required to remove the roots after a few years [48].

The limitation of this systematic review is that most of the studies included are descriptive rather than investigational or analytical. The questionable reproducibility of metric measurements for bone loss using OPG limits its use in this context. Where justified, CBCT is considered more practical for quantifying the bone change status distal to the lower second molar in the case of MTMI. Also, heterogeneity in the design, samples, and methods of investigating bone loss was noticed. One of the limitations of our study is that it focused only on radiographic changes in bone status distal to lower second molar in cases of mandibular third molars, rather than the patient’s clinical history. Future researchers are advised to further investigate the confounding factors contributing to bone loss using three-dimensional radiography when justified.

## 5. Conclusions

In this review, we tried to explore the accuracy of OPG for the evaluation of bone loss distal to the lower second molar. In this respect, panoramic radiography is still considered valuable but not accurate in visualizing mandibular impacted teeth and associated pathologies. Marginal bone loss distal to the lower second molar is visualized clearly by OPG. The metric measurements, however, are inconsistent because of the inherited characteristics of OPG. Since CBCT carries more radiological risks than OPG, the use of CBCT is justified in cases where the benefits of the investigation and the risks that would occur are considered.

## Figures and Tables

**Table 1:** Assessment using the JBI checklist for prevalence study appraisal.

Checklist	Studies							
	Días et al. [40]	Matzen et al. [41]	Gupta et al. [42]	Tai et al. [43]	Savitri et al. [44]	Ye et al. [45]	Altan et al. [46]	Shumar et al. [47]
Was the sample frame appropriate to address the target population?	YES	YES	* UN	YES	YES	YES	YES	YES
Were study participants sampled in an appropriate way?	YES	UN	UN	UN	UN	UN	UN	UN
Was the sample size adequate?	YES	YES	UN	YES	UN	UN	UN	UN
Were the study subjects and the setting described in detail?	YES	YES	YES	YES	YES	YES	YES	YES
Was the data analysis conducted with sufficient coverage of the identified sample?	YES	YES	YES	YES	YES	YES	YES	YES
Were valid methods used for the identification of the condition?	YES	YES	YES	YES	YES	YES	YES	YES
Was the condition measured in a standard, reliable way for all participants?	YES	YES	YES	YES	YES	YES	YES	YES
Was there appropriate statistical analysis?	YES	YES	YES	YES	YES	YES	YES	YES
Was the response rate adequate, and if not, was the low response rate managed appropriately?	YES	YES	YES	YES	YES	YES	YES	YES

* UN = unclear.

**Table 2:** Data extraction and analysis.

Author & Year	Diagnostic Tool	Study Design	Study Type	Sample Size	Bone Loss%	QAT	Rob
Días et al. 2020 [40]	OPG and CBCT	Retrospective	Observational–analytical	70 patients 124 MTMI *	OPG 62.9% CBCT 80%	JBI critical appraisal checklist	Low 100%
Matzen et al. 2017 [41]	OPG and CBCT	Retrospective	Observational–analytical	379 MTMI	OPG 66% CBCT 85%	JBI critical appraisal checklist	Low 88%
Gupta et al. 2020 [42]	OPG	Retrospective	Prevalence	400 patients	39%	JBI critical appraisal checklist	Moderate 66%
Tai et al. 2020 [43]	OPG	Retrospective	Prevalence	2650 MTMI	35.30%	JBI critical appraisal checklist	Low 88%
Savitri et al. 2020 [44]	OPG	Retrospective	Prevalence	53 OPGs	33.3%	JBI critical appraisal checklist	Moderate 77%
Ye et al. 2021 [45]	Clinical and OPG	Retrospective	Prevalence	262 patients 432 MTMI	14.81%	JBI critical appraisal checklist	Moderate 77%
Altan et al. 2019 [46]	OPG	Retrospective	Prevalence	954 patients 1598 MTMI	4.9%	JBI critical appraisal checklist	Moderate 77%
Shumar et al. [47]	OPG	Retrospective	Prevalence	1900 OPGs	6.8%	JBI critical appraisal checklist	Moderate 77%

*, Mandibular Third Molar Impaction.

## Data Availability

All relevant data are provided within the article.
